# Supplementary material for: GT‐rich promoters can drive RNA pol II transcription and deposition of H2A.Z in African trypanosomes
Source: EMBO J. 2017 Jul 12;36(17):2581–94. doi: 10.15252/embj.201695323 (PMC5579346; doi:10.15252/embj.201695323)
Supplement: Supplementary file 1 — Appendix [file EMBJ-36-2581-s001.pdf]

GT-rich promoters can drive RNA pol II transcription and deposition of H2A.Z in African trypanosomes

Carolin Wedel, Konrad U. Förstner, Ramona Derr and T. Nicolai Siegel

Appendix

Table of Contents

Appendix Materials and Methods **2**
Generation of TSR translocation constructs 2
Integration PCR 3
Identification of 10mers enriched on the coding strand of TSR 3
Generation of constructs containing GT-rich promoter 3
RT-qPCR analysis 4
Endogenous RNA pol II and RNA pol I transcription levels 5
Meta-plot generation 5
Dinucleotide analysis of nucleosomal DNA 5
Appendix Figures **6**
Appendix Figure S1 - Characterization of α-H2A.Z antibody................................ 6
Appendix Figure S2 - Sequences of GT-rich elements and their respective reverse complement. 7

Appendix Materials and Methods

Generation of TSR translocation constructs

All cloning reactions were performed using InFusion® HD Cloning Plus reagents according to the manufacturer's instructions (Clontech Laboratories). The targeting construct pCW24v2 (Fig 2B) originates from pLEW100v5_HYG (kind gift from George Cross, Addgene plasmid #24012). To generate pCW24v2, the rRNA spacer targeting sequence of pLEW100v5_HYG was removed by digestion with AlwNI and AflII and replaced with a linker sequence (amplified with TAATCCTGTTACCAGTGGCTGCTGCCAGTGGCGATAAGTC and CTCGAGCTGCATTAATGAATCGGCCAAC from the parental construct) and the upstream homology region Tb427_01_v4:282931-283210 (amplified with TTAATGCAGCTCGAGGCGGCCGCCTACTCACCTGAGAAGCGGC and TCCGGATAGGCTTAAGCATCGTTGGGTGGCAAAGTG from gDNA). To insert the downstream homology region Tb427_01_v4:283239-283591 (amplified with AGGCATGCAAGCTAGCCGAAACCAAGGCGGAAGAAA and AGAGGATCTGGCTAGGCGGCCGCGCATGCGTAGGACTCGGTAT from gDNA), the plasmid was digested with NheI. Prior to transfection, the plasmid was linearized with NotI.

To generate the no-promoter control (pCW24v2-p), pCW24v2 was digested with BglII. The T7 promoter, which transcribes the selection marker, was reinserted with the following sequence: TCTTCTGAAAACGCGTAATACGACTCACTATAGGGCGCGTTTCCTTACAT. The constructs containing TSR DNA sequences were generated by replacing the rRNA promoter in pCW24v2 by BglII digestion and insertion of fragments amplified using different sets of oligonucleotides (listed in Table EV1).

The two TSRs analyzed in our luciferase assay are representative of other TSRs found in the *T. brucei* genome. To avoid partial translocations of genes, which could lead to secondary effects, we selected TSRs that did not contain genes spanning their boundaries. In addition, we chose TSRs lacking NotI and XhoI restriction sites that were required plasmid linearization prior to the transfection.

To generate the targeting construct pCW27v2 (Fig EV1A, upper panel), the upstream and downstream regions of homology of pCW24v2 were removed with XhoI/AflII and NheI, respectively and replaced by the new upstream homology region Tb427_09_v4:1,067,215-1,067,647 (amplified from gDNA with ATTAATGCAGCTCGAGAAGTCAGAAGGGGAAAGCGG and TCCGGATAGGCTTAAGGTTGTACTGGGAGAGGGTGC) and the new downstream homology region Tb427_09_v4:1,067,679-1,068,160 (amplified from gDNA with AGGCATGCAAGCTAGCCGCGCGCATCTCAAATCTAC and AGAGGATCTGGCTAGGCGGCCGCGGGTGCTTGCCTTTCATCAC). Prior to transfection, the plasmid was linearized with NotI and XhoI.

To generate the targeting construct pCW28v2 (Fig EV1A, lower panel), the upstream and downstream regions of homology of pCW24v2 were removed with XhoI/AflII and NheI, respectively and replaced by the new upstream homology region Tb427_10_v5:1,926,616-1,927,048 (amplified from gDNA with ATTAATGCAGCTCGAGCTTTCAGCAAGCACGCAGAG and TCCGGATAGGCTTAAGCGGGAAGAGGTGGTGAACTT) and the new downstream homology region Tb427_10_v5:1,927,082-1,927,505 (amplified from gDNA with GGCATGCAAGCTAGCGGTTCCCTGTGCATAATTCGC and AGAGGATCTGGCTAGGCGGCCGCTCCTCCCATAAATGTACAGCTCG). Prior to transfection, the plasmid was linearized with NotI and XhoI.

The respective no-promoter controls (pCW27v2-p, pCW28v2-p), constructs containing TSR DNA sequences and GT-rich promoters have been generated just as described for pCW24v2.

In order to introduce the targeting constructs pCW27v2 and pCW28v2 and their derivatives into ΔH3.V cells the resistance marker was exchanged and the constructs were labelled with v3. The Hygromycin resistance gene was removed with MscI and SpeI and replaced by a phleomycin resistance gene amplified with ACAGAACAATTTTGGCCACACAACCCGGTGTTAGGATCTCCGAGGCCTTTAGTC CTGCTCCTCGGCC and AAGCTCTAGAACTAGTATGGCCAAGTTGACCAGTGC.

To be able to control for cell number, a renilla luciferase reporter gene was inserted in the VSG pseudogene located in the active 221 bloodstream expression site using pCJ25ARluc (kind gift of Christian Janzen). All constructs were introduced into a *T. brucei* SM cell line.

Integration PCR

To verify the proper integration of the different constructs, integration PCRs on the 5´ end and the 3´ end of the inserted construct were performed with the following oligonucleotides:

pCW24v2: TCGATCTCTGTCAGCAGTCTGTCCTT and CTTGGGCGCAGGGTCGAT (expected product size: 1191 bp), GCTGAATTGGAATCGATATTGTTACA and GAGGAGAACTGCGATGACCC (expected product size: 1912 bp);

pCW27v2: CCAGGAGGGTTTTTACCTGTTGGAAGGG and CTTGGGCGCAGGGTCGAT (expected product size: 1273 bp), GCTGAATTGGAATCGATATTGTTACA and GTATATCTCTCTTTGCATCGCGG (expected product size: 1950 bp);

pCW28v2: TGCCCGCATAACGAGTGGGCG and CTTGGGCGCAGGGTCGAT (expected product size: 1415 bp), GCTGAATTGGAATCGATATTGTTACA and CACAATCCCAAAAATCTAGCACCA (expected product size: 1779 bp);

pCW27v3: CCAGGAGGGTTTTTACCTGTTGGAAGGG and CCTGGCCTGGGTGTGGGT (expected fragment size: 1296 bp), GCTGAATTGGAATCGATATTGTTACA and GTATATCTCTCTTTGCATCGCGG (expected product size: 1950 bp);

pCW28v3: TGCCCGCATAACGAGTGGGCG and CCTGGCCTGGGTGTGGGT (expected fragment size: 1438 bp), GCTGAATTGGAATCGATATTGTTACA and CACAATCCCAAAAATCTAGCACCA (expected product size: 1779 bp).

Identification of 10mers enriched on the coding strand of TSR

To identify 10mers enriched on the coding strand compared to the noncoding strand across TSRs, the sequence of each TSR (n = 199) was divided in 5 equally spaced regions. For each region the distribution of 10mers was determined for the coding and noncoding strand. Those, which are 6-fold enriched in the coding strand compared to the non-coding strand, were counted and plotted.

Generation of constructs containing GT-rich promoter

The synthetic GT promoters (GT_210_nt, GT_206_nt, GT_416_nt) were synthesized by Integrated DNA Technologies (IDT) and are composed of 10mer motifs enriched in the coding strand (listed in Dataset EV1). The 10mers were ordered so that they met synthesis requirements set by IDT and, where necessary, As and Cs were inserted between 10mers to reduce the GT content and to allow synthesis.

All cloning reactions were performed using InFusion® HD Cloning Plus reagents according to the manufacturer's instructions. To generate pCW24v2_GT_210_nt, pCW24v2 was digested with BglII and the synthesized GT_210_nt sequence containing an AscI and an FseI restriction site at the 3´ end was introduced. Digestion with AscI and FseI and insertion of the synthesized GT_206_nt sequence fused both synthesized sequence elements creating pCW24v2_GT_416_nt.

To generate pCW24v2_GT_210_nt_rc, the reverse complement sequence of GT_210_nt was amplified with GTATTAATCAAGATCTAACAACAACCCTCCACACAC and CCGGCCGTAGGCGCGCCGTGTGTGTGGTGCTTTTT and inserted into pCW24v2_GT_210_nt after BglII and AscI digestion.

To generate pCW24v2_GT_416_nt_rc, the reverse complement sequence of GT_416_nt was amplified with GTATTAATCAAGATCTACAAACAAACACAAAAA and TGATAGGGAGATCGGCCGGCCGTGTGTGTGGTGCTTTT and inserted into pCW24v2_GT_416_nt after BglII and FseI digestion. All sequences are listed in Table EV2. Integration PCRs were performed as described for pCW24v2.

The cell lines used in Fig EV6 were generated with pCW24v4 and its derivatives. To generate pCW24v4 (rRNA promoter control) and pCW24v4-p (no promoter control), pCW24v2 was digested with KpnI and SmaI and the synthesized rRNA_promoter sequence and no-promoter sequence were introduced. Both, the rRNA promoter sequence and no-promoter sequence are preceded by two tetracyclin operators.

To generate pCW24v4_GT_210_nt, pCW24v4 was digested with BglII and SmaI and the synthesized GT_210_nt sequence containing an AscI and an FseI restriction site at the 3´ end was introduced. Digestion of the plasmid with AscI and FseI followed by insertion of the synthesized GT_206_nt sequence joined both synthesized sequence elements to create the plasmid pCW24v4_GT_416_nt.

To generate pCW24v4_GT_210_nt_rc, the reverse complement sequence of GT_210_nt was amplified with GTATTAATCAAGATCTTCCCTATCAGTGATAGAGATCTCCCTATCAGTGATAGAG ATAACAACAACCCTCCACACAC and CCGGCCGTAGGCGCGCCGTGTGTGTGGTGCTTTTT and inserted into pCW24v4_GT_210_nt after BglII and AscI digestion.

To generate pCW24v4_GT_416_nt_rc, the reverse complement sequence of GT_416_nt was amplified with GTATTAATCAAGATCTTCCCTATCAGTGATAGAGATCTCCCTATCAGTGATAGAG ATTCTACAAACAAACACAAAAA and CCGGCCGTAGGCGCGCCCCGTGTGTGTGGTGCTTTT and inserted into pCW24v4_GT_210_nt after BglII and AscI digestion. All sequences are listed in Table EV2. Integration PCRs were performed as described for pCW24v2.

RT-qPCR analysis

Total RNA was isolated from 5 x 10^7 cells using the NuclepSpin® RNA kit from Macherey-Nage. 1 μg RNA was used for cDNA synthesis using M-MLV reverse transcriptase (ThermoScientific). The cDNA was diluted 1:8 and RT-qPCR was performed in triplicates using oligos listed in Dataset EV4 and iTaq™ Universal SYBR® Green Supermix (Bio-Rad) in a CFX96™ Touch Real-Time PCR Detection System (Bio-Rad). Genes were chosen based on the wild type expression level and the distance from the insertion site: Tb427.01.890 and Tb427.01.990 are the closest

genes with a distance of 5 kb, each. Tb427.01.860 and Tb427.01.1050 are 17 kb and 22 kb apart, respectively.

Endogenous RNA pol II and RNA pol I transcription levels

To measure endogenous RNA pol II levels (Figure EV4A) pCW37 was generated to target *FLUC* to a locus within a PTU on chromosome 1 (Tb427_01_v4:500,640-501,239). In pCW24v2-p the hygromycin resistance gene was inverted by digestion with SmaI and BglII and insertion of the amplicon of TTGTTAGCAGCATTTAAATCCCGTACCGGGGGCACA and ACTGATAGGGAGATCTTTGCAGAATACTGCATAGATAACAAACGC on pCW24v2-p. The upstream homology region (Tb427_01_v4:500,640-500,939, amplified with ATTAATGCAGCTCGAGCCCTCTGTTTTCACCTCCTCC and TCCGGATAGGCTTAAGAACGAGGAGGAGGGCAAAAG) was exchanged by digestion with XhoI and AflII and the downstream homology region (Tb427_01_v4:500,940-501,239, amplified with AGGCATGCAAGCTAGCAATTTCTCCACCTGTTTCACACT and TCTGGCTAGGCGGCCGCTCACTTGCTTTCACTTCTTCACTTC) by digestion with NheI and NotI. Prior to transfection, the plasmid was linearized with NotI and XhoI.

To measure endogenous RNA pol I transcription levels (Figure EV4B) *FLUC* was targeted to a random rRNA spacer by transfecting Rluc positive cells with NotI-linearized pLEW100v5_HYG.

Meta-plot generation

To average the sequencing data for multiple regions COVERnant's subcommand extract was used. For the generation of meta-plots the mean without zeros of the generated matrices was plotted to the indicated locations.

Dinucleotide analysis of nucleosomal DNA

Sequencing data were processed as already described. From ChIP-seq alignment files in BAM format read pairs that span a genomic region of 147 bp were extracted and the dinucleotide frequencies for all four A/T and all four C/G permutations were determined in those stretches. The frequencies were plotted relative to the nucleosome dyad.

Appendix Figures

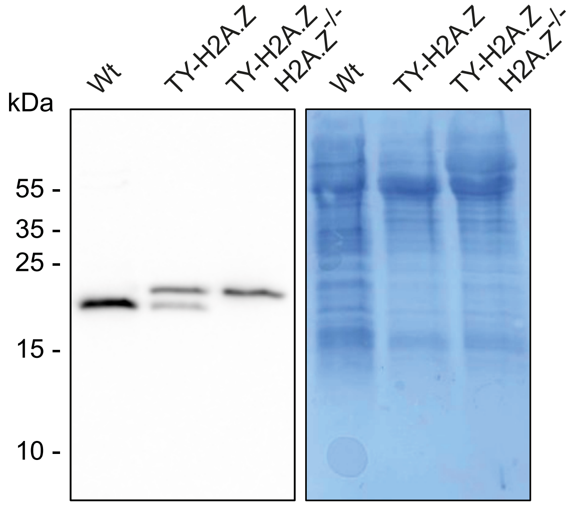

Appendix Figure S1 - Characterization of α-H2A.Z antibody.
Western blot of whole-trypanosome extracts (2 x 10^6 cells per lane). Amido black stained nitrocellulose is shown as loading control (right panel). The antibody was used at a dilution of 1:1000.

GT_210nt

GTGTGTGTGGTGCTTTTTTCGTCTTTTTTTGTGTGTGGGGCGAAGAAAATGTTTGTTTGTTCTTTCTCCCGTGTG
TGTGCTTCCCCCTTTGTGCGTGCGTAGGGGGAGAGTTCCCCCTTTGGGGGGAAACTGTGTGTGGGGTTTGTTTGT
GTGGGTGCGGGGGGGGAAACTTTGTTTTGTCGGTGGTGTTGTGTGTGGAGGGTTGTTGTT

GT_210nt_rc

AACAACAACCCTCCACACACAACACCACCGACAAAACAAAGTTTCCCCCCCCGCACCCACACAAACAAACCCCAC
ACACAGTTTCCCCCCAAAGGGGGAACTCTCCCCCTACGCACGCACAAAGGGGGAAGCACACACACGGGAGAAAGA
ACAAACAAACATTTTCTTCGCCCCACACACAAAAAAAGACGAAAAAAGCACCACACACAC

GT_416nt

GTGTGTGTGGTGCTTTTTTCGTCTTTTTTTGTGTGTGGGGCGAAGAAAATGTTTGTTTGTTCTTTCTCCCGTGTG
TGTGCTTCCCCCTTTGTGCGTGCGTAGGGGGAGAGTTCCCCCTTTGGGGGGAAACTGTGTGTGGGGTTTGTTTGT
GTGGGTGCGGGGGGGGAAACTTTGTTTTGTCGGTGGTGTTGTGTGTGGAGGGTTGTTGTTGGCGCGCCGCGTGTG
TGTAATGTTTTTTGGGGGGGGAGTTTATTTTTTGGTGGGTGGTGGTGTGTGTGTGCGTGCGTGTTTTGTTTTTGG
GGGGGGTTTCCCCCTTTGTTGCTGCTGTTTTTTTGTTCCCCCTCCCCTGTGTGTGTGTGTGTTTGTGCCCTTTTCT
TGTTTGTGTGTCCCCCTCCCCTGTTTTTTGTGTTTGTTTGT

GT_416nt_rc

ACAAACAAACACAAAAAAACAGGGGAGGGGGACACACAAACAAGAAAAGGGCACAAACACACACACACACAGGGGA
GGGGGAACAAAAAAACAGCAGCAACAAAGGGGGAAACCCCCCCAAAAACAAAACACGCACGCACACACACACCAC
CACCCACCAAAAAATAAACTCCCCCCCCAAAAAACATTACACACACGCGGCGCGCCAACAACAACCCTCCACACA
CAACACCACCGACAAAACAAAGTTTCCCCCCCCGCACCCACACAAACAAACCCCACACACAGTTTCCCCCCAAAG
GGGGAACTCTCCCCCTACGCACGCACAAAGGGGGAAGCACACACACGGGAGAAAGAACAAACAAACATTTTCTTC
GCCCCACACACAAAAAAAGACGAAAAAAGCACCACACACAC

Appendix Figure S2 - Sequences of GT-rich elements and their respective reverse complement.
