# Supplementary material for: GT‐rich promoters can drive RNA pol II transcription and deposition of H2A.Z in African trypanosomes
Source: EMBO J. 2017 Jul 12;36(17):2581–94. doi: 10.15252/embj.201695323 (PMC5579346; doi:10.15252/embj.201695323)
Supplement: Supplementary file 2 — Expanded View Figures PDF [file EMBJ-36-2581-s002.pdf]

Expanded View Figures

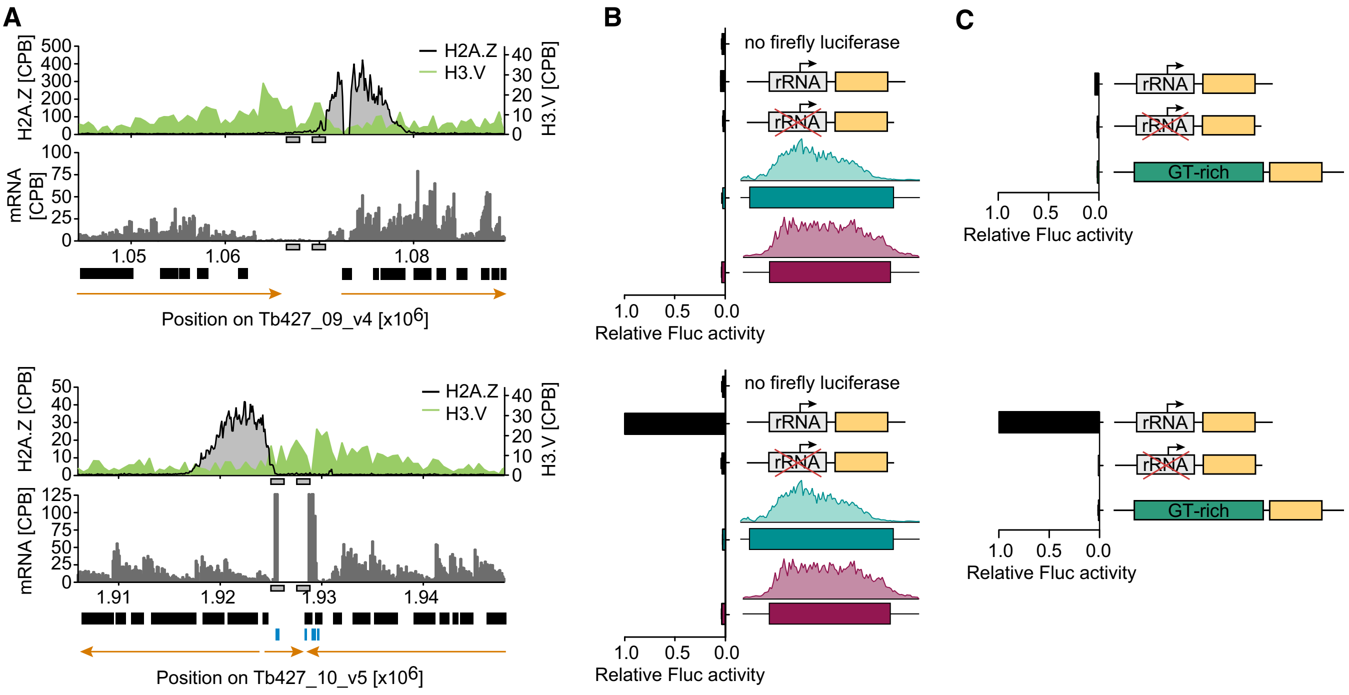

Figure EV1. No promoter activity was detected at sites enriched in H3.V.

A Illustration of reporter target sites with H3.V enrichment (upper panel: between a TTR and TSR of chr. 9; lower panel: between RNA pol III-transcribed genes of chr. 10). Black boxes represent ORFs, and orange arrows indicate the direction of transcription. Gray and blue boxes represent regions of homology and tRNA genes, respectively.

B Luciferase assays were performed after insertion of two different complete TSR DNA sequences or control constructs into the regions indicated in (A). To normalize for differences in cell number, Fluc activity was normalized to ectopically expressed *Renilla* luciferase activity. rRNA promoter-driven Fluc activity in the upper panel was very low. Thus, to normalize for technical variations, values were normalized to rRNA-promoter control values measured in the lower panel.

C Luciferase assays were performed after insertion of GT_210_nt or control constructs into the regions indicated in (A) in a Δ*H3.V* strain. To normalize for differences in cell number, Fluc activity was normalized to ectopically expressed *Renilla* luciferase activity.

Data information: In (B, C), data are presented as mean ± SD. Error bars indicate standard deviation between two replicates.

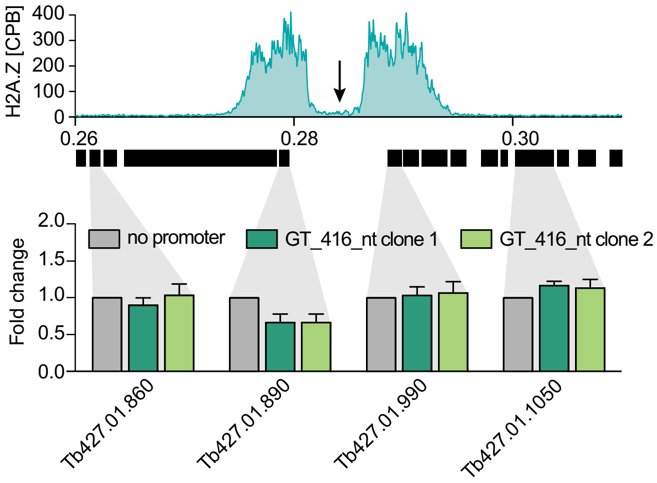

Figure EV2. Impact of GT-rich element insertion on the transcription of the flanking PTUs.

Transcript levels of genes flanking the site of promoter insertion were determined by RT–qPCR after insertion of the GT_416_nt element and after insertion of the same construct lacking a promoter element. Transcript levels after insertion of the promoter-less construct were set to 1 (gray bars), and the relative fold change in transcript levels after insertion of the GT_416_nt element is shown as green bars. Data are presented as mean ± SD. Raw Ct values are listed in Dataset EV4. Error bars indicate standard deviation among triplicates. The black arrow marks the site of reporter insertion.

© 2017 The Authors

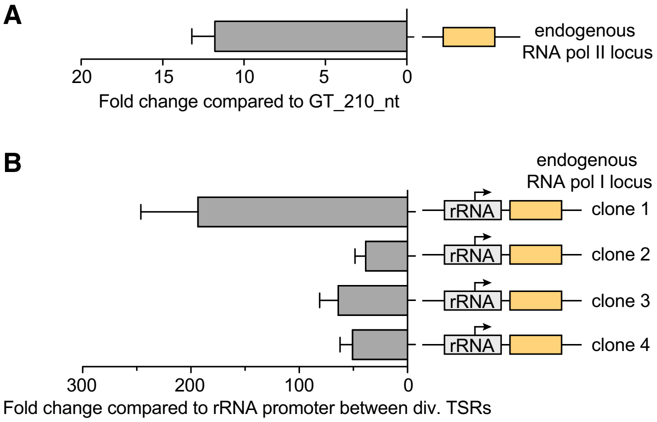

Figure EV3. Endogenous expression levels.

A Luciferase activity expressed from an endogenous RNA pol II locus within a PTU compared to luciferase activity induced by GT_210_nt between dTSRs. To normalize for differences in cell number, Fluc activity was normalized to ectopically expressed *Renilla* luciferase activity.

B Luciferase activity induced by an rRNA promoter at an endogenous RNA pol I locus within the rDNA spacer region compared to the luciferase activity induced by an rRNA promoter located between dTSR. To normalize for differences in cell number, Fluc activity was normalized to ectopically expressed *Renilla* luciferase activity.

Data information: Data are presented as mean ± SD. Error bars indicate standard deviation between two replicates.

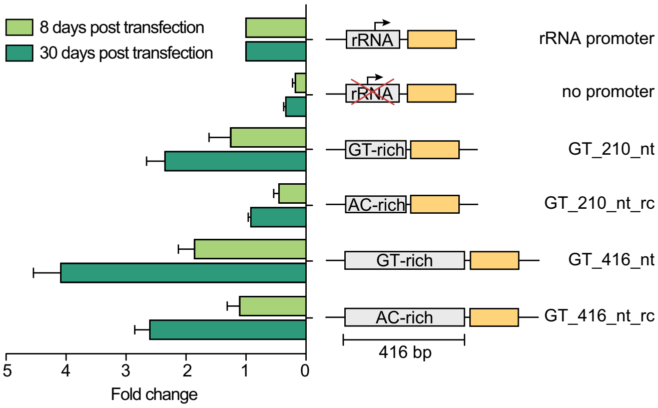

Figure EV4. Luciferase activity increases over time.
Luciferase assays were performed 8 and 30 days post-transfection. To account for differences in cell number, Fluc activity was normalized to ectopically expressed *Renilla* luciferase activity. To account for technical variations, values were normalized to rRNA promoter-driven Fluc activity. Data are presented as mean ± SD. Error bars indicate standard deviation between two replicates.

 © 2017 The Authors

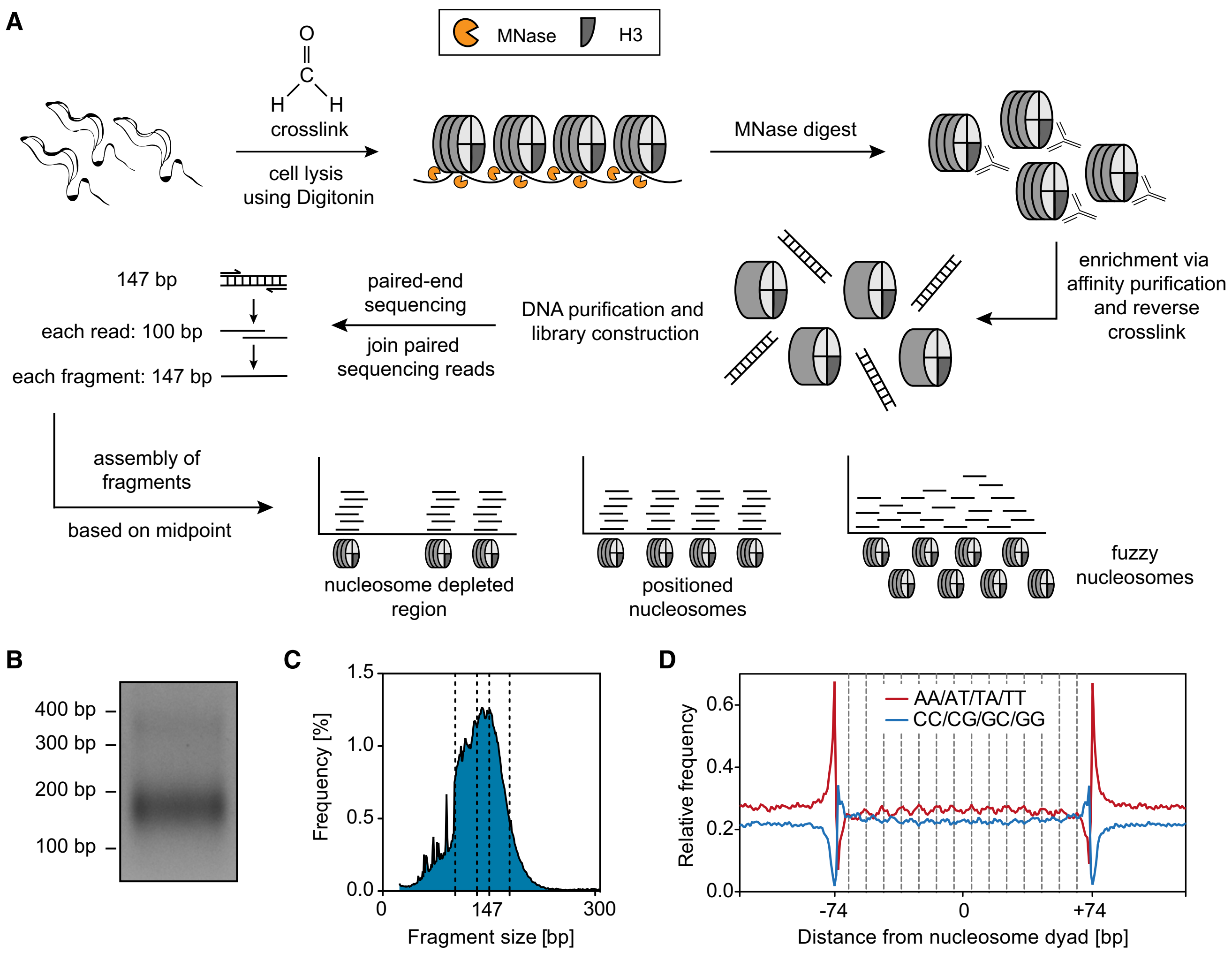

Figure EV5. Establishment of a high-resolution MNase-ChIP-seq protocol for *Trypanosoma brucei*.

A Outline of MNase-ChIP-seq. *T. brucei* cells were formaldehyde-cross-linked and permeabilized, and chromatin was digested into mononucleosomes using MNase. Nucleosomes containing histone H3 were isolated via affinity purification using rabbit H3 antiserum. After reversing cross-links, the nucleosomal DNA was purified and paired-end-sequenced using Illumina HiSeq 2500. The sequencing reads were joined to fragments and assembled according to their midpoints.

B 2% agarose gel with 100 ng of mononucleosomal DNA after an MNase digest.

C Fragment size distribution after sequencing and joining of paired sequencing reads. Dashed lines indicate the fragment sizes 100, 137, 147, and 157 bp.

D Relative frequencies of AA/AT/TA/TT and CC/CG/GC/GG dinucleotides throughout 147 bp of nucleosomal DNA for each bp relative to the nucleosome dyad. Dashed lines indicate distance of 10 bp from position −74 bp.

© 2017 The Authors
