# Supplementary material for: GT‐rich promoters can drive RNA pol II transcription and deposition of H2A.Z in African trypanosomes
Source: EMBO J. 2017 Jul 12;36(17):2581–94. doi: 10.15252/embj.201695323 (PMC5579346; doi:10.15252/embj.201695323)
Supplement: Supplementary file 7 — Table EV1 [file EMBJ-36-2581-s007.pdf]

Table EV1: List of TSR translocation constructs, the source coordinates of the translocated sequences and oligonucleotides for amplification and insertion.

construct	source coordinates	forward primer sequence 5´-3´	reverse primer sequence 5´-3´
pCW24v2_regA	Tb427_10_v5:800,949-810,167	CCGGTACGGGAGATCTCCCTATAGTGAGTCGTATTAATCAAGTTTAGCCCGTTTACCTCCA	ACTGATAGGGAGATCTAGCTCAGCAGTAATAAGGGTCA
pCW24v2_regA1	Tb427_10_v5:800,949-802,743	CCGGTACGGGAGATCTCCCTATAGTGAGTCGTATTAATCAAGTTTAGCCCGTTTACCTCCA	ACTGATAGGGAGATCTGCGGACACGGATTAGCTGAA
pCW24v2_regA2	Tb427_10_v5:802,251-804,045	CCGGTACGGGAGATCTCCCTATAGTGAGTCGTATTAATCAAAAGGCGTATGTCCACTGGG	ACTGATAGGGAGATCTAGGTGTAAGGAAAAACTGAATGAGA
pCW24v2_regA3	Tb427_10_v5:803,552-805,345	CCGGTACGGGAGATCTCCCTATAGTGAGTCGTATTAATCAGGCCCTTTGGTTACCCACTT	ACTGATAGGGAGATCTTCCCACCGTGAGTTAAACCAG
pCW24v2_regA4	Tb427_10_v5:804,859-806,648	CCGGTACGGGAGATCTCCCTATAGTGAGTCGTATTAATCACAAAATTATGGTGCACGTACGGT	ACTGATAGGGAGATCTTACTTCTAGGTGGGGCTCCC
pCW24v2_regA5	Tb427_10_v5:806,150-807,942	CCGGTACGGGAGATCTCCCTATAGTGAGTCGTATTAATCAGCAACGTCTCCTTCGCTCTT	ACTGATAGGGAGATCTAGCCAAGAGGTTTGTGGTTCA
pCW24v2_regA6	Tb427_10_v5:807,455-809,249	CCGGTACGGGAGATCTCCCTATAGTGAGTCGTATTAATCAGGCACCCAAACTGCTGAAAG	ACTGATAGGGAGATCTGCAAAATGCATACGCTCGGT
pCW24v2_regA7	Tb427_10_v5:808,751-	CCGGTACGGGAGATCTCCCTATAGTGAG	ACTGATAGGGAGATCTAGCTCAGCAGTA

	810,167	TCGTATTAATCACGAGGCTTTTGCTAAGAGGGT	ATAAGGGTCA
pCW24v2_regA2rc	Tb427_10_v5:802,251-804,045	CCGGTACGGGAGATCCCCTATAGTGAGTCGTATTAATCAAGATCTAGGTGTAAGGAAAAACTGAATGAGA	ACTGATAGGGAGATCTAAAGGCGTATGTCCACTGGG
pCW24v2_regB	Tb427_10_v5:1,634,960-1,641,653	CCGGTACGGGAGATCTCCCTATAGTGAGTCGTATTAATCACCCGGAAAGTGATGAGGGAG	ACTGATAGGGAGATCTAAAACAATATTTTTCTTCGTCAGCGT
pCW24v2_regB1	Tb427_10_v5:1,634,960-1,636,762	CCGGTACGGGAGATCTCCCTATAGTGAGTCGTATTAATCACCCGGAAAGTGATGAGGGAG	ACTGATAGGGAGATCTCTTATCTGTCCACCAATAGAGTATTTC
pCW24v2_regB2	Tb427_10_v5:1,636,267-1,638,057	CCGGTACGGGAGATCTCCCTATAGTGAGTCGTATTAATCAGAACGCTAACCCCTCCTCG	ACTGATAGGGAGATCTGTGGGACAAACACGGTCACT
pCW24v2_regB3	Tb427_10_v5:1,637,571-1,639,366	CCGGTACGGGAGATCTCCCTATAGTGAGTCGTATTAATCAGGAGTACTAAAGTGCTGCGGA	ACTGATAGGGAGATCTGCAAAGAAGACCATTCGTCAACA
pCW24v2_regB4	Tb427_10_v5:1,638,867-1,640,655	CCGGTACGGGAGATCTCCCTATAGTGAGTCGTATTAATCATACTCCTTTTGCTTGCGGCG	ACTGATAGGGAGATCTCACTCATTTCATAACCGGTCCG
pCW24v2_regB5	Tb427_10_v5:1,640,194-1,641,653	CCGGTACGGGAGATCTCCCTATAGTGAGTCGTATTAATCATTCGTTCTTTGATCAAAAGTGTACGT	ACTGATAGGGAGATCTAAAACAATATTTTTCTTCGTCAGCGT
pCW24v2_regB1rc	Tb427_10_v5:1,636,267-1,638,057	GTATTAATCAAGATCTCTTATCTGTCCACCAATAGAGTATTTC	ACTGATAGGGAGATCTCCCGGAAAGTGATGAGGGAG

underscored sequences indicate InFusion overhangs
