# Supplementary material for: GT‐rich promoters can drive RNA pol II transcription and deposition of H2A.Z in African trypanosomes
Source: EMBO J. 2017 Jul 12;36(17):2581–94. doi: 10.15252/embj.201695323 (PMC5579346; doi:10.15252/embj.201695323)
Supplement: Supplementary file 8 — Table EV2 [file EMBJ-36-2581-s008.pdf]

Table EV2: List of synthesized DNA sequences to generate constructs containing GT-rich promoters or their reverse complement sequence.

**sequence name**	**DNA sequence 5´-3´**
**pCW24v2 background**	
GT_210_nt	GTATTAATCAAGATCTGTGTGTGTGGTGCTTTTTTCGTCTTTTTTTGTGTGTGGGGCGAAGAAAATGTTTGTTTGT<br>TCTTTCTCCCGTGTGTGTGCTTCCCCCTTTGTGCGTGCGTAGGGGGAGAGTTCCCCCTTTGGGGGGAAACTGTG<br>TGTGGGGTTTGTTTGTGTGGGTGCGGGGGGGGAAACTTTGTTTTGTCGGTGGTGTTGTGTGTGGAGGGTTGTTG<br>TTGGCGCGCCTACGGCCGGCCGATCTCCCTATCAGT
GT_206_nt	GTTGTTGTTGGCGCGCCGCGTGTGTGTAATGTTTTTTGGGGGGGGAGTTTATTTTTTGGTGGGTGGTGGTGTGT<br>GTGTGCGTGCGTGTTTTGTTTTTGGGGGGGGTTTCCCCCTTTGTTGCTGCTGTTTTTTGTTCCCCCTCCCCTGTG<br>TGTGTGTGTGTTTGTGCCCTTTTCTTGTTTGTGTGTCCCCCTCCCCTGTTTTTTGTGTTTGTTTGTGGCCGGCCGA<br>TCTCCCTATCA
fused GT_416_nt	GTGTGTGTGGTGCTTTTTTCGTCTTTTTTTGTGTGTGGGGCGAAGAAAATGTTTGTTTGTTCTTTCTCCCGTGTGT<br>GTGCTTCCCCCTTTGTGCGTGCGTAGGGGGAGAGTTCCCCCTTTGGGGGGAAACTGTGTGTGGGGTTTGTTTGT<br>GTGGGTGCGGGGGGGGAAACTTTGTTTTGTCGGTGGTGTTGTGTGTGGAGGGTTGTTGTTGGCGCGCCGCGTG<br>TGTGTAATGTTTTTTGGGGGGGGAGTTTATTTTTTGGTGGGTGGTGGTGTGTGTGTGCGTGCGTGTTTTGTTTTT<br>GGGGGGGGTTTCCCCCTTTGTTGCTGCTGTTTTTTGTTCCCCCTCCCCTGTGTGTGTGTGTGTTTGTGCCCTTTT<br>CTTGTTTGTGTGTCCCCCTCCCCTGTTTTTTGTGTTTGTTTGT
GT_210_nt_rc	GTATTAATCAAGATCTAACAACAACCCTCCACACACAACACCACCGACAAAACAAAGTTTCCCCCCCCGCACCCA<br>CACAAACAAACCCCACACACAGTTTCCCCCCAAAGGGGGAACTCTCCCCCTACGCACGCACAAAGGGGGAAGC<br>ACACACACGGGAGAAAGAACAAACAAACATTTTCTTCGCCCCACACACAAAAAAAGACGAAAAAAGCACCACACA<br>CACGGCGCGCCTACGGCCGG
GT_416_nt_rc	GTATTAATCAAGATCTACAAACAAACACAAAAAACAGGGGAGGGGGACACACAAACAAGAAAAGGGCACAAACA<br>CACACACACACAGGGGAGGGGGAACAAAAAACAGCAGCAACAAAGGGGGAAACCCCCCCCAAAAACAAAACAC<br>GCACGCACACACACACCACCACCCACCAAAAAATAAACTCCCCCCCCAAAAAACATTACACACACGCGGCGCGC

	CAACAACAACCCTCCACACACAACACCACCGACAAAACAAAGTTTCCCCCCCCGCACCCACACAAACAAACCCCACACACAGTTTCCCCCCAAAGGGGGAACTCTCCCCCTACGCACGCACAAAGGGGGAAGCACACACACGGGAGAAAGAACAAACAAACATTTTCTTCGCCCCACACACAAAAAAAGACGAAAAAAGCACCACACACACGGCCGGCCGATCTCCCTATCA
**pCW24v4 background**	
rRNA_promoter	AGTCGTATTAATCAGGTACCTCCCTATCAGTGATAGAGATCTCCCTATCAGTGATAGAGATTAGCTTTCCACCCAGCGCGGGTGCATTCTGGCTCTTATATATACTTATTGTCATGACAGAGTATATTGTACTGTGTTGATAAGGGACGGGTAACTGTATTGAAGAGCCGATGCTTTTGACATGTTAGATATAATATGTTTTATTGTAAAGTCAATACAACACACAATAGGATAATAATGATAAAGTTAAAAAAGTATATATAGTAATAGAAATATATCTTATATAGGAAAGATTAAGCAGTAAAAGTAGCGCTTACGGCGTACGGTCCCTGAGTACTGAGTTTAACATGTTCTCGTCCCGGGCTGCACGCGCCT
no_promoter	AGTCGTATTAATCAGGTACCTCCCTATCAGTGATAGAGATCTCCCTATCAGTGATAGAGATTAGCCCTGAGTACTGAGTTTAACATGTTCTCGTCCCGGGCTGCACGCGCCT
GT_210_nt	GCCCCCGGTACGGGAGATCCCCTATAGTGAGTCGTATTAATCAAGATCTTCCCTATCAGTGATAGAGATCTCCCTATCAGTGATAGAGATGTGTGTGTGGTGCTTTTTTCGTCTTTTTTTGTGTGTGGGGCGAAGAAAATGTTTGTTTGTTCTTTCTCCCGTGTGTGTGCTTCCCCCTTTGTGCGTGCGTAGGGGGAGAGTTCCCCCTTTGGGGGGAAACTGTGTGTGGGGTTTGTTTGTGTGGGTGCGGGGGGGGAAACTTTGTTTTGTCGGTGGTGTTGTGTGTGGAGGGTTGTTGTTGGCGCGCCTACGGCCGGCCTGAGTACTGAGTTTAACATGTTCTCGTCCCGGGCTGCACGCGCCT
GT_206_nt	GTTGTTGTTGGCGCGCCGCGTGTGTGTAATGTTTTTTGGGGGGGGAGTTTATTTTTTGGTGGGTGGTGGTGTGTGTGTGCGTGCGTGTTTTGTTTTTGGGGGGGGTTTCCCCCTTTGTTGCTGCTGTTTTTTGTTCCCCCTCCCCTGTGTGTGTGTGTGTTTGTGCCCTTTTCTTGTTTGTGTGTCCCCCTCCCCTGTTTTTTGTGTTTGTTTGTGGCCGGCCTGAGTACTGAGTT
fused GT_416_nt	GTGTGTGTGGTGCTTTTTTCGTCTTTTTTTGTGTGTGGGGCGAAGAAAATGTTTGTTTGTTCTTTCTCCCGTGTGTGTGCTTCCCCCTTTGTGCGTGCGTAGGGGGAGAGTTCCCCCTTTGGGGGGAAACTGTGTGTGGGGTTTGTTTGTGTGGGTGCGGGGGGGGAAACTTTGTTTTGTCGGTGGTGTTGTGTGTGGAGGGTTGTTGTTGGCGCGCCGCGTGTGTGTAATGTTTTTTGGGGGGGGAGTTTATTTTTTGGTGGGTGGTGGTGTGTGTGTGCGTGCGTGTTTTGTTTTTGGGGGGGGTTTCCCCCTTTGTTGCTGCTGTTTTTTGTTCCCCCTCCCCTGTGTGTGTGTGTGTTTGTGCCCTTTTCTTGTTTGTGTGTCCCCCTCCCCTGTTTTTTGTGTTTGTTTGT

GT_210_nt_rc	GTATTAATCAAGATCTTCCCTATCAGTGATAGAGATCTCCCTATCAGTGATAGAGATAACAACAACCCTCCACACA<br>CAACACCACCGACAAAACAAAGTTTCCCCCCCCGCACCCACACAAACAAACCCCACACACAGTTTCCCCCCAAA<br>GGGGGAACTCTCCCCCTACGCACGCACAAAGGGGGAAGCACACACACGGGAGAAAGAACAAACAAACATTTTC<br>TTCGCCCCACACACAAAAAAAGACGAAAAAAGCACCACACACACGGCGCGCCTACGGCCGG
GT_416_nt_rc	GTATTAATCAAGATCTACAAACAAACACAAAAAACAGGGGAGGGGGACACACAAACAAGAAAAGGGCACAAACA<br>CACACACACACAGGGGAGGGGGAACAAAAAACAGCAGCAACAAAGGGGGAAACCCCCCCCAAAAACAAAACAC<br>GCACGCACACACACACCACCACCCACCAAAAAATAAACTCCCCCCCCAAAAAACATTACACACACGCGGCGCGC<br>CAACAACAACCCTCCACACACAACACCACCGACAAAACAAAGTTTCCCCCCCCGCACCCACACAAACAAACCCC<br>ACACACAGTTTCCCCCCAAAGGGGGAACTCTCCCCCTACGCACGCACAAAGGGGGAAGCACACACACGGGAGA<br>AAGAACAAACAAACATTTTCTTCGCCCCACACACAAAAAAAGACGAAAAAAGCACCACACACACGGCCGGCCGA<br>TCTCCCTATCA

underscored sequences indicate InFusion overhangs
